# A Neutral Model of Transcriptome Evolution

**DOI:** 10.1371/journal.pbio.0020132

**Published:** 2004-05-11

**Authors:** Philipp Khaitovich, Gunter Weiss, Michael Lachmann, Ines Hellmann, Wolfgang Enard, Bjoern Muetzel, Ute Wirkner, Wilhelm Ansorge, Svante Pääbo

**Affiliations:** **1**Max-Planck-Institute for Evolutionary AnthropologyLeipzigGermany; **2**WE Informatik, BioinformatikUniversity of Düsseldorf, DüsseldorfGermany; **3**European Molecular Biology LaboratoryHeidelbergGermany

## Abstract

Microarray technologies allow the identification of large numbers of expression differences within and between species. Although environmental and physiological stimuli are clearly responsible for changes in the expression levels of many genes, it is not known whether the majority of changes of gene expression fixed during evolution between species and between various tissues within a species are caused by Darwinian selection or by stochastic processes. We find the following: (1) expression differences between species accumulate approximately linearly with time; (2) gene expression variation among individuals within a species correlates positively with expression divergence between species; (3) rates of expression divergence between species do not differ significantly between intact genes and expressed pseudogenes; (4) expression differences between brain regions within a species have accumulated approximately linearly with time since these regions emerged during evolution. These results suggest that the majority of expression differences observed between species are selectively neutral or nearly neutral and likely to be of little or no functional significance. Therefore, the identification of gene expression differences between species fixed by selection should be based on null hypotheses assuming functional neutrality. Furthermore, it may be possible to apply a molecular clock based on expression differences to infer the evolutionary history of tissues.

## Abstract 2

Analysis of differences in gene expression between primate species suggests that the majority of them are selectively neutral and likely to have little or no functional consequences

## Introduction

Advances in microarray technology have made the systematic study of expression levels of thousands of transcripts possible. This has been heralded as a major step forward in understanding the function of genomes, since transcript expression levels are expected to correlate with biological functions. Although this is clearly the case for many genes that change their expression in response to environmental stimuli (e.g., Spellman et al. 1998; Hughes et al. 2000; Miki et al. 2001), it is not known whether evolutionary changes in gene expression are determined primarily by Darwinian selection or by stochastic processes. Indeed, the extent to which natural selection has shaped the properties of organisms has been hotly debated ever since Charles Darwin proposed that organisms are adapted to their environment as a result of natural selection. At the molecular level, the view that most changes are due to Darwinian selection was challenged by Kimura's neutral theory of molecular evolution (Kimura 1983). This theory states that the vast majority of differences seen in nucleotide and amino acid sequences within and between species have no or only minor selective effects. Consequently, their occurrence within a species and the fixation of differences between species are primarily the result of stochastic processes. Thus, it is believed today that the evolution of the overwhelming majority of synonymous nucleotide changes within protein-coding exons, as well as changes in noncoding parts of genomes, are determined by mutational processes and random genetic drift (Li 1997). In fact, even at the level of morphology, it has been argued that many features are not adaptive, but instead result from physical constraints or historical accidents (Gould and Lewontin 1979). However, since selection acts at the level of the phenotype while variation is generated at the level of the genotype, the proportion of changes caused by selection can be expected to be largest at the phenotypic level and smallest at the DNA sequence level. As a corollary, we may expect the proportion of selected changes to gradually decrease at the proteome and the transcriptome levels, since these are located progressively further from the phenotype. Consequently, a large proportion of transcriptome changes might be explained by historical accidents rather than by selective events.

To test whether this may be the case, we have investigated whether a neutral model can describe transcriptome differences observed among primate and mouse species as well as among various brain regions within a species.

## Results/Discussion

### Transcriptome Evolution among Species

**Figure 1:**
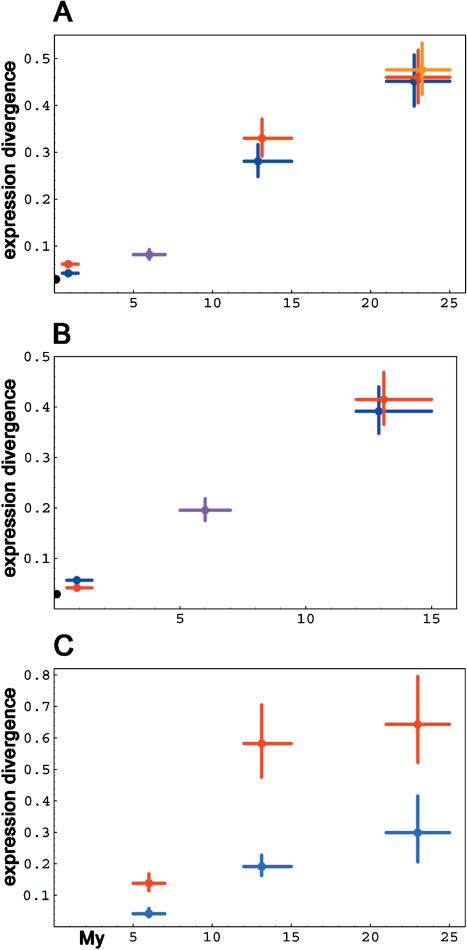
Brain and Liver Transcriptome Change among Primates as a Function of Time Average expression differences within and between primates in brains (A), in liver (B), and for genes in brain for genes with high (red) and low (blue) variation among six humans (C). Colors: red, comparisons between and with humans; blue, comparisons between and with chimpanzees; purple, comparisons between humans and chimpanzees; orange, comparisons between orangutan and rhesus macaque; black, comparisons between experimental duplicates. Vertical error bars for expression indicate 95% confidence intervals calculated by 10,000 bootstraps over genes. Divergence times are according to Glazko and Nei (2003).

If the majority of evolutionary changes are caused by historical accidents rather than by natural selection, they will accumulate mainly as a function of time rather than as a function of morphological or behavioral change of organisms. Applied to transcriptome evolution, a neutral model therefore implies that the rate of transcriptome change is proportional to time. In particular, if we assume that mutations cause changes in the relative amounts of transcripts independently of the absolute expression level of the gene, then the squared difference of the logarithm of the expression level is expected to increase linearly with divergence time (Lande 1976; Felsenstein 2004). To investigate whether this is the case, we have studied differences in the gene expression levels of around 12,000 genes in the prefrontal cortex of six humans, three chimpanzees *(Pan trogodytes),* one orangutan *(Pongo pygmaea),* and one rhesus macaque *(Macaca mulatta)* using oligonucleotide microarrays. To exclude the influence of DNA sequence differences on the hybridization results, at least between humans and chimpanzees, only oligonucleotide probes that matched perfectly to the chimpanzee DNA sequences were used in the analysis (see Materials and Methods). In Figure 1A, we plot species divergence times against the average squared difference between the logarithm of the expression levels of 1,998 genes that had expression levels large enough to be detected in all primate samples. Although comparisons involving orangutan and rhesus were complicated by nucleotide sequence differences to array probes, the result shows that the squared differences represent an approximately linear function of time over at least 20 million years. When we apply the same analysis to published gene expression data for the livers of three humans, three chimpanzees, and one orangutan (Enard et al. 2002), we again observe a linear relationship between gene expression differences and species divergence times (Figure 1B).

**Figure 2:**
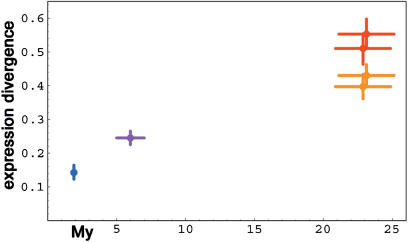
Brain Transcriptome Change as Measured by cDNA Arrays Colors and symbols as in Figure 1 except orange, which indicates comparisons between chimpanzee and both macaque species, and blue, which indicates comparisons between rhesus macaque and crab-eating macaque. Divergence times are according to Hayasaka et al. (1996) and Glazko and Nei (2003).

Since oligonucleotide-based microarrays are sensitive to DNA sequence differences and the orangutan and rhesus macaque genome sequences are not yet known—so that we cannot delete oligonucleotides carrying mismatches between the species—we used arrays containing around 28,000 cDNAs ranging in length from 500 to 1,500 nucleotides to assay gene expression patterns in the prefrontal cortex of six humans, five chimpanzees, five rhesus macaques, and five crab-eating macaques *(Macaca fascicularis).* Due to the greater probe length, these arrays are much less sensitive to DNA sequence differences and therefore can be used to compare gene expression in humans and macaques (Ranz et al. 2003). When we plot the extent of gene expression divergence for 5,829 genes whose expression was detected in all samples against species divergence time, we again observe that expression differences accumulate approximately linearly with time (Figure 2).

In a recent study of gene expression in the brains of humans, chimpanzees, and orangutans, we found that the rate of expression change on the human lineage has been larger than on the chimpanzee lineage (Enard et al. 2002). This is in apparent contradiction to the linearity observed here. However, the analysis of Enard et al. (2002) was based on less than 5% of all genes expressed in the brain because it was confined to genes that differed significantly in expression between humans and chimpanzees. In contrast, here we perform a transcriptome-wide analysis of all genes with detectable expression in several primate species. However, the slightly higher divergence of humans than chimpanzees from the two macaque species may reflect the previously reported higher rate of gene expression divergence on the human evolutionary lineage (Enard et al. 2002; Caceres et al. 2003; Gu and Gu 2003). However, additional experiments are necessary to exclude the possibility that this is caused by experimental artifacts.

The clocklike accumulation of expression differences between species observed for primates is in agreement with the recent observation that differences in gene expression are consistent with phylogenetic relationships among *Drosophila* species (Rifkin et al. 2003), and both these observations are compatible with the predictions of the neutral model. However, under certain selection scenarios, positively selected changes would also accumulate linearly with time (Felsenstein 2004). Therefore, linear accumulation of expression differences alone does not rule out selection.

In addition to the clocklike accumulation of evolutionary changes, the neutral theory states that the same forces determine the rate of evolution both within and between species (Kimura 1983). Thus, a neutral prediction with respect to transcriptome evolution is that genes that vary more within species should be more likely to change between species as well. In order to test this, we ranked 2,926 genes with detectable expression levels in six humans and three chimpanzees according to their variation within humans and calculated the species divergences for the 25% of genes that had the largest and the smallest human variation, respectively. Figure 1C shows that the genes with high variation among humans changed significantly faster between species than the genes with low variation. The magnitude of observed expression differences may be influenced by DNA sequence mismatches affecting hybridization between orangutan and rhesus samples and array probes. However, the difference in divergence rates between genes with high and low expression variation within species is unlikely to be explained by hybridization artifacts, since this would require a difference in sequence divergence between the two groups of genes.

We further considered the correlation between the average diversity within humans and chimpanzees and the divergence between the species for the 2,926 genes. This correlation is highly significant (*p* < 0.001) as gauged by a permutation test (see Materials and Methods). Since all array probes that carried sequence differences between humans and chimpanzees were removed prior to analysis, this correlation is not affected by hybridization artifacts. The strength of the correlation (τ = 0.24) is of a similar magnitude as the one obtained for the correlation of diversity and divergence of random genomic DNA sequences in humans and chimpanzees (τ = 0.179, *p* = 0.028, *n* = 76), the vast majority of which are noncoding (Hellmann et al. 2003). Thus, although the two measures are not directly comparable, the degree of correlation between intraspecific diversity and interspecific divergence is similar for brain transcriptomes and random genomic DNA sequences in humans and chimpanzees.

**Figure 3:**
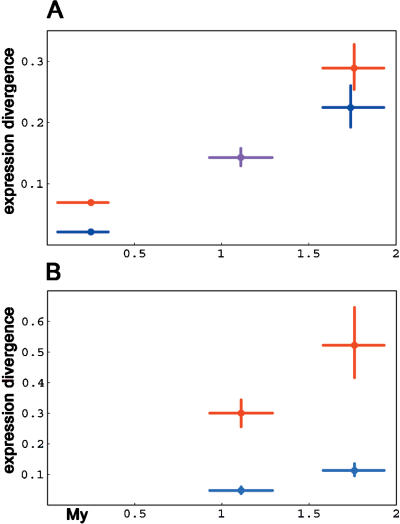
Brain Transcriptome Change among Mice as a Function of Time Average expression differences within and between the mouse species (A) and for genes with high (red) and low (blue) variation among M. musculus individuals (B). Colors: red, comparisons between and with *M. musculus;* blue, between and with *M. spretus;* purple, between M. musculus and M. spretus. Vertical error bars for expression indicate 95% confidence intervals calculated by 10,000 bootstraps over genes. Divergence times are according to She et al. (1990).

To investigate whether gene expression differences accumulate as a function of time also in another group of mammals, we analyzed three mouse species. An advantage in this case is that post mortem artifacts are less likely to influence the results than in the case of autopsy material of humans and great apes. We determined differences in gene expression levels for around 9,000 genes in the frontal cortex of six outbred *Mus musculus,* three outbred *M. spretus,* and one *M. caroli.* As shown in Figure 3A, the squared transcriptome differences accumulated linearly with time among the mouse species. To test if divergence rates differ for the genes with high and low variation within species, we investigated the 25% of the 2,742 genes detected in all samples with the highest and the lowest variation within *M. musculus,* respectively, as was done in the primates. Figure 3B shows that genes that vary more within M. musculus diverged faster among mouse species than genes that vary less. As in the case of primate species, imperfect matches of M. spretus and M. caroli mRNAs to the array oligonucleotides may partly influence the observed expression differences between species. Nonetheless, as for primates, the difference in divergence rates between genes with high and low expression variation within species is unlikely to be explained by hybridization differences since there is no indication that genes that vary more in expression within species diverge faster between species with respect to their DNA sequence. The correlation between diversity and divergence for M. musculus and M. spretus for genes detected in both species is highly significant (τ = 0.29, *p* < 0.001, *n* = 3,139), although in this case we cannot correct for DNA sequence differences. A correlation between gene expression differences within and between species was recently demonstrated also in teleost fish (Oleksiak et al. 2002). Thus, in agreement with the neutral model, genes that vary more within species tend to vary more between species in three vertebrate groups.

### A Test for Neutrality

One way to test whether gene expression differences between species accumulate at a rate consistent with neutral expectation is to compare them to the expression differences observed for a class of genes that can reasonably be expected to not be the direct targets of positive or negative selection. Since expressed pseudogenes do not produce any functional gene products, they can be viewed as such a class of genes. Thus, if a substantial proportion of intact genes accumulate expression differences faster than pseudogenes, this would indicate that they are positively selected. Such an observation would falsify a neutral model.

To test this, we considered the expression patterns in four regions of the brain in three humans and three chimpanzees using the Affymetrix U95 array set interrogating approximately 40,000 genes (Philipp Khaitovich, unpublished data). In order to identify all probe sets on these arrays that interrogate expressed pseudogenes, we aligned the probe sequences, as well as published lists of human pseudogenes, to the human genome (see Materials and Methods). In total, 889 probe sets that overlap with pseudogenes were identified. Thirty-three of these were detected (detection *p*-value < 0.05) in at least one of four brain regions in either the chimpanzees or the humans after masking all probes carrying DNA sequence differences between the species. Of these, 28 contained at least one mutation that leads to a loss of function in both humans and chimpanzees. We therefore assumed that these pseudogenes were nonfunctional in the common ancestor of humans and chimpanzees. Finally, we checked whether these probe sets may crosshybridize with any intact genes by aligning them to the human genome. This left us with 23 expressed pseudogenes.

**Figure 4:**
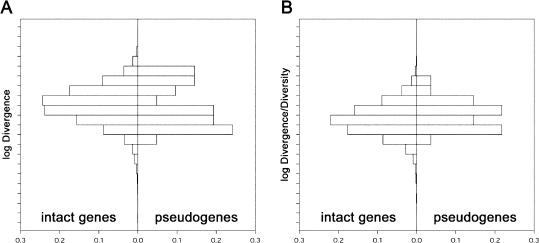
Comparison between Intact Genes and Pseudogenes (A) shows the distributions of expression divergence between humans and chimpanzees for intact genes and pseudogenes. (B) shows the distributions of the ratio of expression divergence between humans and chimpanzees and expression diversity within humans for intact genes and pseudogenes.

We compared the distributions of the squared differences between the mean expression levels of each gene in humans and in chimpanzees for the 23 pseudogenes and 12,647 intact genes for each of the four brain regions. In each case, only the genes detected in a given brain region were used for the calculation. In all four brain regions the distribution of expression distances among intact genes did not differ significantly from that among pseudogenes in either a Kolmogorov-Smirnov test or a Wilcoxon rank sum test. These tests would have been significant if more than 5% (1/23) of the genes had a distribution radically different from that of the pseudogenes. When the data for four brain regions were combined, no visual difference between the two distributions was apparent (*p* = 0.16 and *p* = 0.69, respectively) (Figure 4A).

Thus, we failed to detect any significant excess of intact genes that diverged faster in expression than pseudogenes. This indicates that the fraction of gene expression differences between the species that are fixed by positive selection is small. Interestingly, there was also no detectable excess of intact genes that diverged slower than pseudogenes. This may seem unexpected, since the expression of many intact genes might be thought to be stabilized by negative selection and therefore to change more slowly than pseudogenes. This may indicate that purifying selection as well is a weak force affecting gene expression. However, it should be noted that the small number of expressed pseudogenes analyzed limits the power to detect positive and negative selection. A targeted effort to study expressed pseudogenes in closely related species would be a worthwhile undertaking.

### A Test for Positive Selection

The fact that the overall accumulation of expression differences conforms to a selectively neutral model does not mean, of course, that all expression differences between species are selectively neutral. As for nucleotide changes, some changes in gene expression will have had phenotypic consequences and some of these will have become fixed due to positive selection. To identify such gene expression differences, we propose to use the ratio of divergence between species to diversity within species, akin to the tests suggested for quantitative genetic traits (Charlesworth 1984; Lynch and Hill 1986; Turelli et al. 1988) and in agreement with recent suggestions by Rifkin et al. (2003) or Hsieh et al. (2003). However, to do this it is necessary for each gene considered to distinguish the gene expression diversity caused by genetic differences between individuals from the diversity caused by environmental factors. This is crucial since the environmental component is likely to be much larger than the genetic component. For example, under strict neutrality and no environmental influence, we expect a divergence to diversity ratio that is equal to the ratio of time of divergence of the species to the average time to the common ancestors of the individuals sampled within a species. This would be about 1:10 for humans and chimpanzees (Chen and Li 2001; Lander et al. 2001). However, the observed ratio is approximately 1:3, suggesting that the environmental component is on the order of three times bigger than the genetic component. Studies of gene expression differences among individuals with different genetic relatedness will eventually allow an estimation of the genetic component of expression variation.

Since we are unable to tease apart genetic and environmental contributions to expression diversity, we instead used pseudogenes to estimate the distribution of divergence to diversity ratios observed in the absence of selection and compared these ratios to intact genes. No significant difference was found (Kolmogorov-Smirnov test, *p* = 0.388; Wilcoxon rank sum test, *p* = 0.134), and both distributions appeared to center around roughly the same values (Figure 4B). Note that this observation has to be taken cautiously since it is based on a small number of pseudogenes and the gene expression diversity is calculated from only three human individuals. Nevertheless, this result indicates that there is no drastic difference between the expression patterns of intact genes and expressed pseudogenes, since our tests would have been significant if 5% or more of the genes had had a radically different divergence to diversity ratio than that observed among the pseudogenes.

### Transcriptome Evolution among Brain Regions

**Figure 5:**
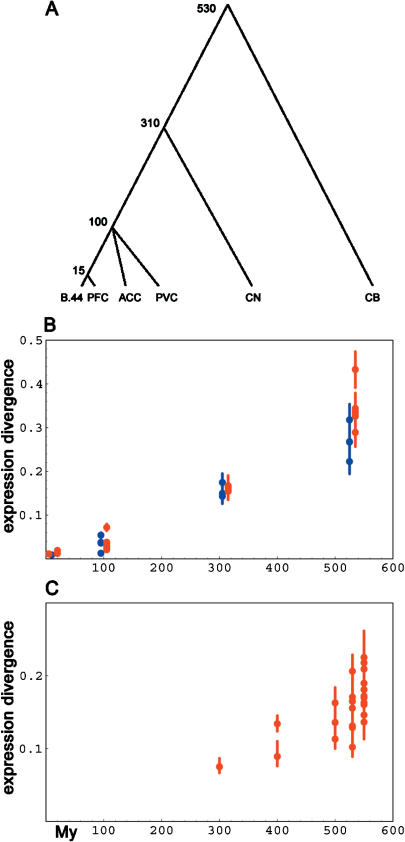
Transcriptome Change among Brain Regions as a Function of Evolutionary Time (A) Schematic evolutionary tree for six human brain regions: B.44, Brodmann's area 44; PFC, prefrontal cortex; ACC, anterior cingulate cortex; PVC, primary visual cortex; CN, caudate nucleus; and CB, cerebellum. Numbers indicate approximate divergence time in millions of years (Butler and Hodos 1996; Nieuwenhuys et al. 1998). (B) Average expression differences among brain regions in humans (red) and in chimpanzees (blue). (C) Average expression differences among brain regions in *M. musculus.* Error bars for expression indicate 95% confidence intervals calculated from 10,000 bootstrap replications over genes.

Different anatomical brain structures appeared at different times during vertebrate evolution. These time points can be viewed as divergence times between brain regions extending millions of years back in the past (Figure 5A). If gene expression changes between different brain regions have a large random component, gene expression differences between brain regions within species could potentially be used as a molecular clock to time the divergences of tissues. To investigate whether this may be the case, we compared expression patterns for Brodmann's area 44, the prefrontal cortex, the anterior cingulate cortex, the primary visual cortex, the caudate nucleus, and the cerebellum in three adult human and three adult chimpanzee males (Philipp Khaitovich, unpublished data). All comparisons were performed between brain regions within the same individual. This has two advantages. First, such comparisons are unaffected by nucleotide sequence variation between and within species. Second, environmental differences and post mortem changes have little effect when expression differences within one individual are studied. In Figure 5B, we plot the average squared distances between the six brain regions in humans and chimpanzees against the time when these brain regions emerged during vertebrate evolution (Butler and Hodos 1996; Nieuwenhuys et al. 1998) for 2,297 and 2,525 genes detected in all human and all chimpanzee samples, respectively. It can be seen that the expression differences increase approximately linearly with time over more than half a billion years. To investigate if this finding holds also in another mammalian species, we used published expression data for 1,346 genes with detectable expression in eight brain regions in the mouse (Su et al. 2002). In this case as well there is an approximately linear relationship between transcriptome differences and evolutionary divergence times (Figure 5C).

If gene expression differences between the brain regions were largely adaptive, one would expect them to correlate with tissue function and not with evolutionary divergence time. Our data show that tissues that diverged recently have very similar gene expression profiles irrespective of the differences in function. For instance, the transcriptome of Brodmann's area 44 in the left hemisphere (Broca's area) is very similar to that of the prefrontal cortex in both humans and chimpanzees, although it is known to be involved in speech processing in humans while it must have another function in chimpanzees (Kandel et al. 2000). This is what we would expect if the time since divergence rather than the extent of functional differences determined the magnitude of transcriptome change. Thus, although a number of expression differences between brain regions surely correspond to functional differences, our findings suggest that a sizeable proportion of the differences are functionally neutral.

A noteworthy finding is that the accumulation of expression differences between brain regions within a species is much slower than the accumulation of expression differences within a brain region between species. In fact, the expression differences that have accumulated among the primate species over 20 million years (see Figure 1A) are approximately as extensive as those that have accumulated among brain regions over 500 million years (see Figure 5B). This is likely to result from the fact that all expression differences seen between brain regions within an individual are caused by changes in regulatory networks established during development by cells that carry the same genome. In addition, expression differences between brain regions reflect the different cell-type compositions of these regions. In contrast, transcriptome differences between species are the result of changes in regulatory networks and cellular composition of tissues, as well as nucleotide sequence differences between species that affect promoters and other genomic elements that determine transcript levels. Our results show that the latter type of changes are much more common than the former.

A possible alternative explanation for the correlation between differences in gene expression and evolutionary divergence time among brain regions could be that differences in gene expression do not correlate with evolutionary divergence time, but instead with divergence time during fetal development. Our observations would then result from the fact that both developmental divergence times and expression differences correlate with evolutionary divergence. A correlation between developmental and evolutionary divergence times has been hypothesized before (for a review, see Gould 1977). In fact, gene expression analyses now provide a quantitative approach to address this question and may also provide a tool to date the evolutionary emergence of brain regions that cannot be discerned in the fossil record.

## Conclusions

We show that a neutral model of evolution can predict the main features of transcriptome evolution in the brains of primates and mice. A neutral model is also in agreement with published observations in *Drosophila* (Rifkin et al. 2003) and fish (Oleksiak et al. 2002). Although selective scenarios that explain some or even most of these observations can be found, the combined evidence presented leads us to conclude that a neutral model is the most adequate null model for transcriptome evolution. This suggests that the majority of gene expression differences within and between species are not functional adaptations, but selectively neutral or nearly neutral. The main challenge now is to develop a mathematical model of transcriptome evolution that allows quantitative predictions of transcriptome changes. Such a model, combined with experimental data estimating the normal variation of gene expression within a species and the relative contributions of genetic and environmental factors to this variation, should allow adaptive gene expression changes to be identified. Further work is also needed to reveal whether proteome evolution is also dominated by changes that are largely selectively neutral.

Finally, the finding that gene expression differences can be used as a molecular clock to date tissue divergences opens the prospect of reconstructing the evolutionary history of organs and tissues based on gene expression measurements in a single species.

## Materials and Methods

### 

#### Tissue samples and microarray data collection

For the primate samples, approximately 200 mg of gray matter was collected from post mortem brain samples from prefrontal cortex region corresponding to Brodmann's area 9 in the left hemisphere from six male humans who were 45, 45, 63, 65, 70, and 70 years old; five male chimpanzees that were 7, 12, 12, 12, and approximately 40 years old; one 16-year-old male orangutan; five approximately 10-year-old male rhesus macaques; and five approximately 15-year-old male crab-eating macaques. All individuals had no history of brain-related diseases and suffered sudden deaths without associated brain damage. For the mouse samples, approximately 50 mg of gray matter was collected from the frontal cortex regions of six M. musculus (three of which are previously described in Enard et al. 2002), three *M. spretus,* and one M. caroli individuals. All mice were outbred, older than 14 weeks, and healthy. Total RNA was isolated using the TRIzol reagent (GIBCO, San Diego, California, United States) according to manufacturer's instructions and purified with Quiagen RNeasy kit (Quiagen, Valencia, California, United States) following the “RNA cleanup” protocol. RNAs were of high and comparable quality as gauged by the ratio of 28S to 18S ribosomal RNAs visualized on agarose gels and by the signal ratios between the probes for the 3′ and 5′ ends of the mRNAs of GAPDH and β-actin genes used as quality controls on Affymetrix microarrays (Affymetrix, Santa Clara, California, United States).

For Affymetrix microarrays, labeling of 5 μg of the RNA, hybridization, staining, washing steps, and array scanning were carried out following Affymetrix protocols. Expression data were collected using Affymetrix HG U95Av2 arrays for the primate samples and Affymetrix MG U74Av2 arrays for the mice samples. The Affymetrix CEL files containing expression data for the different regions of the mouse brain, including amygdala, cerebral cortex, hippocampus, hypothalamus, cerebellum, olfactory bulb, and two regions of spinal cord were provided by John Hogenesch.

Arrays containing 51,000 cDNAs corresponding to approximately 40,000 UniGene clusters were manufactured in the laboratory of W.A. as described elsewhere (Anonymous 2003). Labeling, hybridization, staining, washing, and array scanning were carried out as described by Cortes-Canteli et al. (2004) with slight modifications. All samples were hybridized twice with dye reversal, using a mixture of all samples as a common reference. All primary expression data were submitted to the Array Express database (http://www.ebi.ac.uk/arrayexpress/).

#### Masking of sequence differences between humans and chimpanzees

In order to exclude all oligonucleotide probes that did not match perfectly between humans and chimpanzees, we aligned all Affymetrix target sequences (http://www.affymetrix.com/analysis/index.affx) first to the human genome (build 33) and then to a draft version of the chimpanzee genome (the assembly was given courtesy of David Jaffe in June 2003). Using BLAT (Kent 2002), we matched chimpanzee sequences with Affymetrix target sequences containing the 16 oligonucleotide probes and determined the best hit using a scoring function. The chimpanzee sequence was then aligned to the human genome to determine whether the best match coincided with the match obtained from alignment of Affymetrix target sequences with the human genome. To identify insertion and deletions (indels), we compared the alignment of the Affymetrix target sequence to the human genome and to the chimpanzee genome, and differences in the indel structure relative to the target sequence were identified as indels. We then identified all oligonucleotide probes within target sequences that matched the chimpanzee sequence perfectly. These probes were used for the analysis while the rest of the probes were masked.

#### Microarray data analysis

Affymetrix microarray image data were analyzed with Affymetrix Microarray Suite v5.0 using default parameters. Arrays were scaled to the same average intensity using all probes on the array. Detected genes were defined as those with a detection *p*-value less than or equal to 0.05. For calculation of the expression values, data were processed with the Bioconductor “affy” software package (Ihaka and Gentleman 1996) using the quantile normalization procedure (Bolstad et al. 2003). cDNA arrays were analyzed using the TM4 software package (Saeed et al. 2003). Detected genes were defined as those with a spot intensity exceeding the background intensity by more than 2-fold. All slides were normalized to the common reference using the LOWESS normalization algorithm. For calculation of diversity and divergence, signal to reference ratio measurements were transformed into standardized signal intensities by multiplying them by the average reference intensity for each gene. Divergence was defined as the squared difference between the mean expression of two groups of samples averaged over (all detected) genes. Diversity was defined as the expression variance within a group of samples.

#### Correlation significance test

We measured the divergence between human and chimpanzee by looking at the squared difference between the mean expression values in humans and chimpanzees. This estimate of divergence includes the errors in our estimates of the two means, which is proportional to the variance in each of the species, and thus to the diversity in each species. Therefore, even if no correlation between divergence and diversity existed, our measured divergence and diversity estimates would correlate, and the smaller the divergence is relative to diversity, the stronger the correlation would be. To estimate if the observed correlation is larger than that expected from this effect alone, we performed a randomization test, in which we computed how much correlation between diversity and divergence would be generated from the above effect even if no correlation between diversity and divergence exists. To be conservative, we first generated a distribution that deliberately underestimated the real divergence between humans and chimpanzees. This was done by first generating a distribution of the expected observed differences (X) in gene expression between humans and chimpanzees if the real divergence is zero. Then using this distribution and the observed distribution of differences (Z), we generated a distribution (Y) that-added to values from X-would give Z. In order to underestimate the divergence, we generated Y assuming that the correlation of X and Y is one. We then generated random samples in the following way: For each gene (g), we chose a random difference of expression (d) from our generated distribution. We then drew six samples from a normal distribution whose mean is zero and whose variance is the diversity in humans for gene g, and three samples from a normal distribution whose mean is d and whose variance is the diversity of chimps for gene g. For these expression values we then calculated the correlation between diversity and divergence. We repeated the whole procedure 1,000 times. None of these randomizations generated a correlation that is as strong as the observed one. To make sure that the whole test is conservative, we generated 100 datasets of three types, all of which had a similar diversity, but had a “real” divergence distribution of (1) zero, (2) the underestimated divergence, or (3) the measured divergence, and had uncorrelated diversity and divergence. We then performed the whole test described above, doing just one randomization test. If the test was not conservative, one would expect the correlation in the dataset to be higher than the correlation after randomization in 50% of the cases. Instead, the correlation after randomization was higher in 98, 98, and 99 cases respectively-showing that our test is indeed conservative.

#### Expressed pseudogenes

We retrieved sequences of all pseudogenes as determined by Torrents et al. (2003), Zhang et al. (2003), and the VEGA project (http://vega.sanger.ac.uk). These sequences, as well as the Affymetrix target sequences, were mapped to the human genome (build 34) using BLAT (Kent 2002), and the best hit was determined using the following parameters: match, +1; mismatch, −3; gap-opening penalty only for gaps ≤ 20, −5; and gap extension, −1. Next, using BLAT, we determined the Affymetrix target sequences where the best-matching sequence did not overlap with the genomic region of a known gene (http://genome.ucsc.edu). Thus, we identified 889 probe sets that overlapped with a pseudogene, but not with a known gene. Combined with gene expression data collected in four brain regions (anterior cingulate cortex, Broca'a area, caudate nucleus, cerebellum; Philipp Khaitovich, unpublished data) in three humans and three chimpanzees, 33 of these probe sets had detectable expression levels in at least one brain region in either three chimpanzees or three humans. For these probe sets, we checked whether at least one of the identified interruptions of the human pseudogene was also present in the chimpanzee, indicating that the pseudogene was already nonfunctional at the time of the chimpanzee–human divergence. This left us with 28 probe sets that were checked for crosshybridization with other genes by aligning oligonucleotide probes from these probe sets to the human genome. Finally, we were left with 23 expressed pseudogenes that did not match perfectly to any other gene by more than seven out of 16 probes in the probe set.
